# GDF5 single-nucleotide polymorphism rs143383 is associated with lumbar disc degeneration in Northern European women

**DOI:** 10.1002/art.30169

**Published:** 2011-03

**Authors:** F M K Williams, M Popham, D J Hart, E de Schepper, S Bierma-Zeinstra, A Hofman, A G Uitterlinden, N K Arden, C Cooper, T D Spector, A M Valdes, J van Meurs

**Affiliations:** 1 King's College LondonLondon, UK; 2 Erasmus Medical CentreRotterdam, The Netherlands; 3 University of OxfordOxford, UK; 4 University of Oxford, Oxford, UK and MRC Epidemiology Resource Centre, University of SouthamptonSouthampton, UK

## Abstract

**Objective:**

Lumbar disc degeneration (LDD) is a serious social and medical problem which has been shown to be highly heritable. It has similarities with peripheral joint osteoarthritis (OA) in terms of both epidemiology and pathologic processes. A few known genetic variants have been identified using a candidate gene approach, but many more are thought to exist. GDF5 is a gene whose variants have been shown to play a role in skeletal height as well as predisposing to peripheral joint OA. In vitro, the gene product growth differentiation factor 5 has been shown to promote growth and repair of animal disc. This study was undertaken to investigate whether the GDF5 gene plays a role in LDD.

**Methods:**

We investigated whether the 5′ upstream single-nucleotide polymorphism (SNP) variant rs143383 was associated with LDD, using plain radiography and magnetic resonance imaging to identify disc space narrowing and osteophytes, in 5 population cohorts from Northern Europe.

**Results:**

An association between LDD and the SNP rs143383 was identified in women, with the same risk allele as in knee and hip OA (odds ratio 1.72 [95% confidence interval 1.15–2.57], *P* = 0.008).

**Conclusion:**

Our findings in 5 population cohorts from Northern Europe indicate that a variant in the GDF5 gene is a risk factor for LDD in women. Many more such variants are predicted to exist, but this result highlights the growth and differentiation cellular pathway as a possible route to a better understanding of the process behind lumbar disc degeneration.

Lumbar disc degeneration (LDD) is a common cause of back pain, which is a major public health problem in Western countries. It is a common cause of work absenteeism, and its prevalence is increasing. LDD is known to be influenced by genetic factors, with estimated heritability of >70%. Little is known, however, of the genetic processes underlying the development of disc degeneration. A number of genetic loci have been investigated through a candidate gene approach. Many early reports of candidate gene associations in LDD, however, have not been replicated (1). LDD has much in common with osteoarthritis (OA) of the knee. The pathologic changes are similar in cartilage and disc and, furthermore, rapid progression of OA of the knee is predicted by LDD (2).

The gene GDF5 is known to be involved in digit growth and certain monogenic disorders of skeletal development, and it was recently identified as playing a role in height (3). There is good evidence that variants in or near the GDF5 gene predispose to knee OA (4–7) and are associated with hip geometry and fracture risk (8). In addition, the gene promotes growth and repair in animal intervertebral disc tissue in vitro (9). The GDF5 gene, therefore, is a good candidate for influencing human intervertebral disc degeneration. The variant rs143383 is found in the 5′-untranslated region of the GDF5 gene on chromosome 20 at position 104 and represents a change of base from T to C. This is the most widely studied single-nucleotide polymorphism (SNP) for GDF5 and, because funding restricted our study to a single SNP, we selected this one to study for association with LDD.

## PATIENTS AND METHODS

### Subjects

We investigated the association between rs143383 and LDD in 5 independent population cohorts from Northern Europe (the Chingford study [10], the Hertfordshire study [11], Rotterdam cohorts 1 and 3 [12], and the TwinsUK registry [13]). All participants provided written consent, and local ethics committee approval had been obtained. Since the pathologic processes, including underlying genes, may differ, disc space narrowing and anterior osteophyte formation were considered separately as well as together.

### Plain radiography of spine

Female participants in the Chingford study (n = 758) underwent plain radiography of the lateral lumbar spine, with LDD scored using a 4-point scale for disc space narrowing and anterior osteophytes over the 4 uppermost lumbar vertebrae. (L5–S1 was not reliably visualized on plain film.) Subjects were classified as having disc space narrowing (narrowing present at ≥2 levels), osteophytes (osteophytes present at ≥2 levels), or a combination (cases of narrowing plus osteophyte formation). Subjects in the Hertfordshire study (n = 336) (http://www.mrc.soton.ac.uk/herts/) underwent radiography and coding of degenerative change as previously described (11). Two different cohorts from the Rotterdam study (Rotterdam cohort 1 [n = 2,577] and Rotterdam cohort 3 [n = 975]) were included. These subjects underwent similar plain radiography and scoring of LDD as previously described (14). Subjects were considered to have LDD if, considering the uppermost 4 lumbar discs, either narrowing was present at ≥2 levels, osteophytes were present at ≥2 levels, or there was a combination of narrowing and osteophytes.

### Magnetic resonance imaging (MRI) of the spine

Female twins (n = 613) from the TwinsUK cohort who had previously undergone T2-weighted sagittal MRI of the lumbar spine were selected. The scans had been coded for degenerative change using a 4-point scale (0–3) for the following features: disc height, disc signal intensity, disc bulge, and anterior osteophyte. The individual traits were investigated, and subjects were considered to be cases if they had a score for a particular trait of ≥2 at 2 or more levels (L1/2 to L5–S1).

### Genotyping

For the Rotterdam cohort, data on genotypes at rs143383 were obtained from the results of a genome-wide association scan using the version 3 Illumina Infinium II HumanHap550 SNP chip array. Genotyping procedures were followed according to the recommendations of the manufacturer (Illumina), as previously described (15). For the Chingford, Hertfordshire, and TwinsUK cohorts, genomic DNA was extracted from peripheral blood leukocytes from affected individuals and controls using standard protocols. Genotyping was carried out by Kbioscience Ltd (Hertfordshire, UK). The rs143383 SNP was genotyped using KASPar chemistry, which is a competitive allele-specific polymerase chain reaction SNP genotyping system using fluorescence resonance energy transfer quencher cassette oligos. Genotyping accuracy, as determined from the genotype concordance between duplicate samples, was 100% (52 samples genotyped in duplicate as a control). The polymorphism was in Hardy-Weinberg equilibrium in controls (*P* > 0.05).

### Statistical analysis

To study the relationship between rs143383 and LDD, a logistic regression model was applied. All analyses were performed with and without adjustment for sex, age, and body mass index (BMI) in each study separately. Meta-analysis of the results in the different cohorts was carried out in the statistical environment R, and forest plots were produced. Heterogeneity was evaluated with the Q statistic and I^2^. If heterogeneity existed (I^2^ >25%), a random-effects model was used for the analysis; otherwise, a fixed-effects model (inverse variance method) was applied. Since many reported associations of genetic variants are not yet replicated, we wished to determine the likelihood of our finding being a true positive, so the false-positive report probability was calculated (16).

## RESULTS

Characteristics of the 5 population samples are shown in Table 1. The total sample size was 5,259. The Chingford cohort consisted entirely of women, and in the TwinsUK group women formed the vast majority. Sex distribution was more equable in the Hertfordshire and Rotterdam studies. With regard to the 2 phenotypes considered, the number of cases was broadly similar for disc space narrowing (range 9–19%) but varied greatly for prevalence of osteophytes (range 6–62%). Both age and BMI (where available) were found to be positively associated in those cohorts having a broad age range, so were adjusted for in the analyses.

**Table 1:** Characteristics of the 5 study groups included in the meta-analysis*

	Rotterdam cohort 1	Rotterdam cohort 3	Chingford study	TwinsUK registry	Hertfordshire birth cohort
Imaging method	Plain radiography	Plain radiography	Plain radiography	T2-weighted MRI	Plain radiography
Sample size	2,577	975	758	613	336
% women	57	58	100	97	39
Age, mean (range) years	65.7 (55–90)	54.7 (48–60)	62.9 (53–77)	53.6 (19–73)	65.8 (61–73)
BMI, mean (range) kg/m^2^	26.3 (16–45)	27.1 (14–57)	26.6 (17–47)	25.0 (16–51)	26.9 (16–40)
Disc space narrowing	490 (19)	102 (10)	68 (9)	108 (17)	50 (15)
Osteophytes	781 (30)	179 (18)	175 (23)	35 (6)	208 (62)
Disc space narrowing plus osteophytes	305 (12)	60 (6)	54 (7)	19 (3)	40 (12)
MAF	39.9	40.4	38.6	34.9	32.3

* Except where indicated otherwise, values are the number (%). MRI = magnetic resonance imaging; BMI = body mass index; MAF = minor allele frequency.

**Figure 1:**
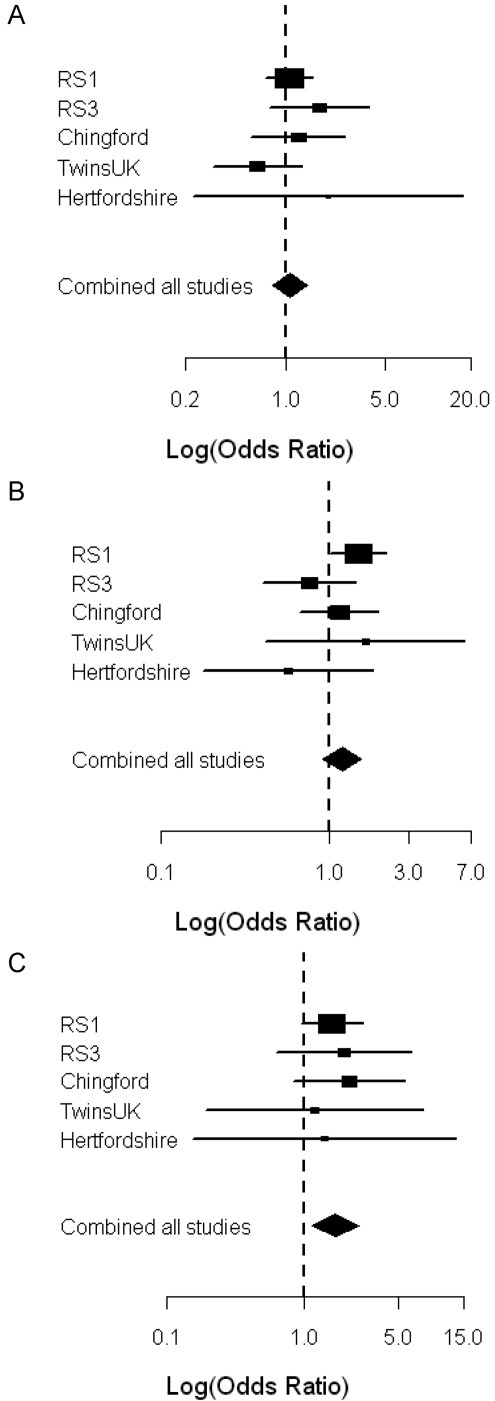
Forest plots of the fixed-effects meta-analysis of the odds ratios (ORs) and 95% confidence intervals (95% CIs) for A, disc space narrowing, B, osteophytes, and C, both disc space narrowing and osteophytes in women in the Rotterdam study 1 (RS1), Rotterdam study 3, Chingford, and TwinsUK cohorts, adjusted for age and body mass index (BMI); in the Hertfordshire cohort, adjusted for age; and in all studies combined, adjusted for age and BMI. Squares represent the ORs for the T allele; bars show the 95% CIs. Diamonds represent the pooled ORs and 95% CIs. The pooled OR for disc space narrowing and osteophytes combined was 1.72 (95% CI 1.15–2.57) (*P* = 0.008).

Minor allele frequencies of rs143383 were broadly similar across the study groups and consistent with that reported in HapMap (C allele 33.3%). Table 2 shows the number of cases and controls with the different alleles of rs143383. The results of the meta-analysis for the association between the T allele and the LDD phenotypes in women, adjusted for age and BMI, are shown in Figure 1. (Results for the total sample are shown in Supplementary Figure 1, available on the *Arthritis & Rheumatism* web site at http://onlinelibrary.wiley.com/journal/10.1002/(ISSN)1529-0131.) A significant association was found with the combination of disc space narrowing plus osteophytes in women, with an odds ratio (OR) of 1.72 (95% confidence interval [95% CI] 1.15–2.57) (*P* = 0.008). Heterogeneity was not detected, so a fixed-effects model was used.

**Table 2:** Genotype distribution for rs143383 in women, by trait and cohort*

	Cases			Controls			
	TT	TC	CC	TT	TC	CC	Total
Disc space narrowing							
Rotterdam cohort 1	114	173	49	420	517	190	1,463
Rotterdam cohort 3	30	22	9	178	244	89	572
Chingford	29	28	11	269	307	114	758
TwinsUK	43	44	17	210	217	58	589
Hertfordshire	7	9	1	56	41	16	130
Osteophytes							
Rotterdam cohort 1	150	177	50	384	512	189	1,462
Rotterdam cohort 3	33	43	20	175	223	78	572
Chingford	69	80	26	229	255	99	758
TwinsUK	17	13	3	236	249	71	589
Hertfordshire	35	30	12	28	20	5	130
Disc space narrowing and osteophytes							
Rotterdam cohort 1	70	103	21	464	586	218	1,462
Rotterdam cohort 3	15	14	4	193	252	94	572
Chingford	25	23	6	273	312	119	758
TwinsUK	9	7	2	246	256	72	592
Hertfordshire	5	8	1	58	42	16	130

* Values are the number of subjects with each genotype.

Distributions of the rs143383 genotypes and ORs unadjusted and adjusted for age are shown for women in each of the cohorts in Supplementary Table 1, available on the *Arthritis & Rheumatism* web site at http://onlinelibrary.wiley.com/journal/10.1002/(ISSN)1529-0131. Using a relatively high prior probability of 20%, the false-positive report probability was calculated, assuming expected effect sizes of OR 1.2 and OR 1.5, for the predisposing T allele. The false-positive report probability is the probability of no true association between a genetic variant and disease given a statistically significant finding, which depends not only on the observed *P* value but also on both the prior probability that the association is real and the statistical power of the test. The false-positive report probability for adjusted values was 36% and 12%, respectively, suggesting that this is not likely to be a false-positive finding.

## DISCUSSION

An association between the GDF5 SNP rs143383 and LDD in women is an important finding and is consistent with the known association of the T allele with OA (17). LDD is common and known to be a cause of low back pain, a condition that results in great social costs. It has been established that LDD is highly heritable, and some genetic variants have been identified (for review, see ref.^1^), but much of the variation remains to be accounted for. The finding of an association with the combination of disc space narrowing and osteophytes, but only in women, may reflect the sample size or may indicate that the effect is detectable only in the most severely affected. Sex-specificity of the effects of genetic variants is well recognized in OA.

GDF5 is a proven variant in peripheral joint OA; this has been demonstrated in a number of populations. Furthermore, it is associated with skeletal height, suggesting mediation by either disc or vertebral body height or long bone length (8). GDF5 was selected as an OA candidate gene initially because phalangoepiphyseal dysplasia patients develop severe, early-onset hip OA. Miyamoto et al (5) performed a case–control association study of a number of SNPs around GDF5 and found the most significant to be rs143383. This association has been shown for both hip and knee OA in both Japanese and Han Chinese populations and, more recently, confirmed in European populations in 2 published meta-analyses (6, 17). Since the T to C mutation at position +104 lies in the promoter region of GDF5, the hypothesis is that the variant influences GDF5 transcription, with individuals with OA having reduced transcriptional ability.

This study has a number of strengths and weaknesses. First, it combines a number of well-characterized cohorts that have been subjected to LDD phenotyping, albeit using different methods. The study of degenerative disease of the disc lags behind the study of hip, knee, and hand OA. In addition, LDD suffers from a lack of standardized epidemiologic definitions, which hampers attempts to study it in a systematic way. The use of twins and any relatedness within the other cohorts was taken into account. This study had 80% power to detect an OR for the T allele of 1.19 or higher for narrowing and 1.15 for osteophytes in the total sample size with *P* < 0.05 (assuming an additive genetic model and T allele frequency of 60%, using Quanto software version 1.2.4). This is further supported by the results of the false-positive report probability, suggesting that the results are more likely to be true positive than false positive.

We investigated whether the 5′-untranslated region SNP rs143383 GDF5 polymorphism was associated with LDD using 5 cohorts of European descent and found evidence of association in women. This SNP is thought to result in reduced transcription of GDF5, a prochondrogenic growth factor which has been shown to increase fiber size in the ligament complex model of knee instability in rats. One might postulate a number of mechanisms by which the SNP exerts its influence on LDD risk, including direct effects on the disc itself or by influence on periarticular structures such as the longitudinal spinal ligaments. Further work is needed to establish its mechanism of action, and this may reveal cellular pathways leading to a greater understanding of the pathophysiology of lumbar disc degeneration.
