# Supplementary material for: GDF5 single-nucleotide polymorphism rs143383 is associated with lumbar disc degeneration in Northern European women
Source: Arthritis Rheum. 2011 Mar;63(3):708–12. doi: 10.1002/art.30169 (PMC3498734; doi:10.1002/art.30169)
Supplement: Supplementary file 1 — Supplementary Figure 1. [file art0063-0708-sd1.doc]

**Supplementary Figure 1.**

| A |  |
| --- | --- |
| B |  |
| C |  |

**Legend to Supplementary Figure 1**

Forest plots of the fixed effect meta-analysis of the odds ratios, adjusted for age and body mass index (except Hertfordshire, age only) for (A) males, (B) females and (C) both genders. Odds ratios (OR, black squares) and 95% CIs (bars) are shown for each study group. The pooled ORs (95% CI) are represented by black diamonds.

RS1 represents Rotterdam Study 1, RS3 Rotterdam Study 3, NAR disc space narrowing, OST anterior osteophytes.
