# Supplementary material for: GDF5 single-nucleotide polymorphism rs143383 is associated with lumbar disc degeneration in Northern European women
Source: Arthritis Rheum. 2011 Mar;63(3):708–12. doi: 10.1002/art.30169 (PMC3498734; doi:10.1002/art.30169)
Supplement: Supplementary file 2 — Supplementary Table 1. [file art0063-0708-sd2.doc]

| **Rotterdam Study 1** | | | | **Rotterdam Study 3** | | | |
| --- | --- | --- | --- | --- | --- | --- | --- |
| NAR | TT | TC | CC | NAR | TT | TC | CC |
| nr cases/ total nr (% cases) | 114/534 (21.3%) | 173/689 (25.1%) | 49/239 (20.5%) | nr cases/ total nr (% cases) | 30/208 (14.4%) | 22/266 (8.3%) | 9/98 (9.2%) |
| OR crude | 1 | 1.24 [0.94-1.62] | 0.95 [0.65-1.38] | OR crude | 1 | 0.54 [0.30-0.96] | 0.60 [0.27-1.31] |
| OR age/BMI adjusted | 1 | 1.30 [0.98-1.72] | 0.94 [0.64-1.39] | OR age/BMI adjusted | 1 | 0.56 [0.31-1.00] | 0.58 [0.26-1.29] |
| OST | TT | TC | CC | OST | TT | TC | CC |
| nr cases/ total nr (% cases) | 150/534 (28.1%) | 177/689 (25.7%) | 50/239 (20.9%) | nr cases/ total nr (% cases) | 33/208 (15.9%) | 43/266 (16.2%) | 20/98 (20.4%) |
| OR crude | 1 | 0.89 [0.69-1.14] | **0.68 [0.47-0.98]** | OR crude | 1 | 1.02 [0.62-1.68] | 1.36 [0.73-2.52] |
| OR age/BMI adjusted | 1 | 0.90 [0.70-1.17] | **0.67 [0.46-0.97]** | OR age/BMI adjusted | 1 | 1.12 [0.67-1.86] | 1.32 [0.70-2.49] |
| NAR + OST | TT | TC | CC | NAR + OST | TT | TC | CC |
| nr cases/ total nr (% cases) | 70/534 (13.1%) | 103/689 (14.9%) | 21/239 (8.8%) | nr cases/ total nr (% cases) | 15/208 (7.2%) | 14/266 (5.3%) | 4/98 (4.1%) |
| OR crude | 1 | 1.17 [0.84-1.62] | 0.64 [0.38-1.07] | OR crude | 1 | 0.72 [0.34-1.52] | 0.55 [0.18-1.70] |
| OR age/BMI adjusted | 1 | 1.21 [0.86-1.69] | 0.62 [0.37-1.05] | OR age/BMI adjusted | 1 | 0.78 [0.37-1.68] | 0.50 [0.16-1.57] |
| **Chingford** | | | | **TwinsUK** | | | |
| NAR | TT | TC | CC | NAR | TT | TC | CC |
| nr cases/ total nr (% cases) | 29/298 (9.7%) | 28/335 (8.3%) | 11/125 (8.8%) | nr cases/ total nr (% cases) | 43/253 (16.9%) | 44/261 (16.8%) | 17/75 (22.7%) |
| OR crude | 1 | 0.85 [0.49-1.46] | 0.90 [0.43-1.85] | OR crude | 1 | 0.99 [0.61-1.61] | 1.43 [0.73-2.81] |
| OR age/BMI adjusted | 1 | 0.88 [0.50-1.54] | 0.81 [0.38-1.72] | OR age/BMI adjusted | 1 | 1.25 [0.75-2.09] | 1.56 [0.77-3.18] |
| OST | TT | TC | CC | OST | TT | TC | CC |
| nr cases/ total nr (% cases) | 69/298 (23.2%) | 80/335 (23.9%) | 26/125 (20.8%) | nr cases/ total nr (% cases) | 17/253 (6.7%) | 13/262 (5.0%) | 3/74 (4.1%) |
| OR crude | 1 | 1.04 [0.72-1.50] | 0.87 [0.52-1.45] | OR crude | 1 | 0.72 [0.34-1.54] | 0.59 [0.16-2.10] |
| OR age/BMI adjusted | 1 | 1.09 [0.74-1.60] | 0.87 [0.51-1.47] | OR age/BMI adjusted | 1 | 0.82 [0.38-1.80] | 0.61 [0.15-2.37] |
| NAR + OST | TT | TC | CC | NAR + OST | TT | TC | CC |
| nr cases/ total nr (% cases) | 25/298 (8.4%) | 23/335 (6.9%) | 6/125 (4.8%) | nr cases/ total nr (% cases) | 9/255 (3.5%) | 7/263 (2.7%) | 2/74 (2.7%) |
| OR crude | 1 | 0.81 [0.45-1.45] | 0.55 [0.22-1.38] | OR crude | 1 | 0.75 [0.26-2.13] | 0.76 [0.16-3.59] |
| OR age/BMI adjusted | 1 | 0.83 [0.45-1.52] | 0.46 [0.18-1.19] | OR age/BMI adjusted | 1 | 1.08 [0.35-3.42] | 0.83 [0.13-5.16] |
| **Hertfordshire** | | | | **Supplementary Table 1: odds ratios for LDD in women by rs143383 genotype in the 5 independent population cohorts**  **Legend to Table 1**  NAR – disc space narrowing  OST – osteophytes  NAR + OST – positive for both disc space narrowing and osteophytes  OR represents odds ratio, nr number, T and C the alleles at rs143383  significant ORs are emboldened | | | |
| NAR | TT | TC | CC |
| nr cases/ total nr (% cases) | 7/63 (11.1%) | 9/50 (18%) | 1/17 (5.9%) |
| OR crude | 1 | 1.76[0.60-5.10] | 0.50 [0.06-4.37] |
| OR age adjusted | 1 | 1.75 [0.60-5.10] | 0.50 [0.06-4.42] |
| OST | TT | TC | CC |
| nr cases/ total nr (% cases) | 35/63 (55.5%) | 30/50 (60.0%) | 12/17 (70.1%) |
| OR crude | 1 | 1.20 [0.57-2.55] | 1.92 [0.60-6.10] |
| OR age adjusted | 1 | 1.22 [0.57-2.62] | 1.75 [0.54-5.64] |
| NAR + OST | TT | TC | CC |
| nr cases/ total nr (% cases) | 5/63 (7.9%) | 8/50 (16%) | 1/17 (5.9%) |
| OR crude | 1 | 2.20 [0.67-7.23] | 0.72 [0.08-6.66] |
| OR age/BMI adjusted | 1 | 2.26 [0.68-7.30] | 0.70 [0.08-6.48] |
